# Association of serum magnesium levels with the risk of spontaneous bacterial peritonitis and sepsis in hospitalized patients with cirrhosis: a retrospective cohort study

**DOI:** 10.3389/fimmu.2026.1857975

**Published:** 2026-09-15

**Authors:** Xiang-Jie Duan, Yang Cai, Qing Su, Qin Zhou, Man Li, Jin-Lian Chen, Chang-Yun Zhang, Duo-Meng Yang, Hai-Yan Yin, Wan-Jie Gu

**Affiliations:** 1 Department of Intensive Care Unit, The First Affiliated Hospital of Jinan University, Guangzhou Guangdong, China; 2 Department of Infectious Diseases, Changde Hospital, Xiangya School of Medicine, Central South University (The First People’s Hospital of Changde City), Changde, China; 3 Department of Emergency, The First Affiliated Hospital of Jinan University, Guangzhou Guangdong, China; 4 Department of Intensive Care Unit, Changde Hospital, Xiangya School of Medicine, Central South University (The First People’s Hospital of Changde City), Changde, China; 5 Department of Pathophysiology, Key Laboratory of State Administration of Traditional Chinese Medicine of the People’s Republic of China, School of Medicine, Jinan University, Guangzhou, Guangdong, China

**Keywords:** cirrhosis, hypomagnesemia, sepsis, serum magnesium, spontaneous bacterial peritonitis

## Abstract

**Background:**

Hypomagnesemia is common in patients with cirrhosis. This study aimed to examine the association between serum magnesium levels and the risks of hospital-acquired spontaneous bacterial peritonitis (HA-SBP) and sepsis.

**Methods:**

This retrospective study included patients with cirrhosis who were hospitalized at the First People’s Hospital of Changde City, China, between May 2021 and June 2025. The exposure variable was the first serum magnesium measurement obtained within 48 hours of admission. The primary outcome was HA-SBP, and the secondary outcome was sepsis. Multivariable regression and restricted cubic spline (RCS) analyses were used to assess the associations.

**Results:**

A total of 1110 patients with cirrhosis were included. The incidences of HA-SBP and sepsis were 12.25% and 6.06%, respectively. Multivariable regression analysis showed that per 1-SD increase in serum magnesium, the risks of HA-SBP and sepsis decreased by 20.8% (odds ratio [OR] = 0.792, 95% confidence interval [CI]: 0.642–0.970, P = 0.026) and 35.5% (OR = 0.645, 95% CI: 0.464–0.883, P = 0.008), respectively. Compared with normomagnesemia, hypomagnesemia was associated with increased risks of HA-SBP (OR = 1.520, 95% CI: 1.009–2.286, P = 0.044) and sepsis (OR = 2.213, 95% CI: 1.189–4.177, P = 0.013). Sensitivity analyses yielded consistent results. RCS analysis showed a linear inverse association between serum magnesium levels and the risks of HA-SBP and sepsis (both P for nonlinearity > 0.05). In addition, the areas under the receiver operating characteristic curves for serum magnesium in predicting HA-SBP and sepsis were 0.635 (95% CI: 0.583–0.686) and 0.688 (95% CI: 0.617–0.758), respectively.

**Conclusions:**

Hypomagnesemia was associated with increased risks of HA-SBP and sepsis in patients with cirrhosis, and these findings should be further validated in prospective studies.

## Introduction

Patients with cirrhosis are prone to spontaneous bacterial peritonitis (SBP) because of portal hypertension, impaired intestinal barrier function, and immune dysfunction (1). The reported prevalence of SBP among hospitalized patients with cirrhosis ranges from 10.81% to 68.20% across different regions (2). SBP is an important trigger of acute decompensation in cirrhosis and is closely associated with increased risks of sepsis, organ failure, and short-term mortality (1). Studies have shown that with the spread of multidrug-resistant bacteria and worsening antimicrobial resistance, the clinical burden and mortality risk of cirrhosis-related SBP continue to increase (3). Since the management of SBP depends on early recognition and timely intervention, identifying early predictors of SBP is of important clinical significance for improving the prognosis of patients with cirrhosis.

Magnesium is the second most abundant intracellular cation in the human body and plays an important role in energy metabolism, protein synthesis, regulation of inflammation, and maintenance of immune homeostasis (4). Serum magnesium is a simple and readily available biomarker that can partly reflect magnesium status (5). However, hypomagnesemia is an easily overlooked electrolyte disorder in hospitalized patients (6). Patients with cirrhosis are more likely to experience magnesium loss or deficiency because of inadequate intake, impaired intestinal absorption, medication use, and renal dysfunction, thereby increasing the risk of hypomagnesemia (7, 8). In addition, studies have shown that serum magnesium levels may further decline with increasing severity of liver disease (9).

Several studies have shown that hypomagnesemia is closely associated with various adverse outcomes in patients with cirrhosis, including acute kidney injury, hepatic encephalopathy, and hepatocellular carcinoma (7, 9–12). However, evidence regarding the relationship between serum magnesium levels and infectious complications such as SBP and sepsis in patients with cirrhosis remains limited (13–15). Given the complex mechanisms underlying cirrhosis-related infections, the association between serum magnesium levels and infection outcomes may be nonlinear. Therefore, this study aimed to evaluate the association between serum magnesium levels and the risks of hospital-acquired spontaneous bacterial peritonitis (HA-SBP) and sepsis in a real-world cohort of hospitalized patients with cirrhosis.

## Materials and methods

### Study population

This retrospective cohort study included adult patients with cirrhosis who were hospitalized at the First People’s Hospital of Changde City, China, between May 2021 and June 2025. The First People’s Hospital of Changde City is a major tertiary teaching hospital in northwestern Hunan Province, China, with a large clinical database of patients with cirrhosis. Patients with cirrhosis were identified using discharge diagnosis codes based on the International Classification of Diseases, Tenth Revision, which have a positive predictive value exceeding 90% (16, 17) (**Supplementary Materials and Method A**). The exclusion criteria were as follows: (1) no serum magnesium measurement within 48 hours after admission; (2) age <18 years; (3) hospital stay < 24 hours; and (4) diagnosis of community-acquired SBP. For patients with multiple hospitalizations, only the first hospitalization was included in the analysis.

This study was conducted in accordance with the ethical principles of the Declaration of Helsinki and was approved by the Ethics Committee of the First People’s Hospital of Changde City (approval number: 2025-332-01). The requirement for informed consent was waived because of the retrospective design and the use of anonymized patient data. This study followed the Strengthening the Reporting of Observational Studies in Epidemiology guidelines (18).

### Exposure variable and outcomes

The exposure variable was the first serum magnesium measurement obtained within 48 hours of admission. Serum magnesium was analyzed both as a continuous variable and as a categorical variable based on the laboratory reference range: hypomagnesemia (<0.75 mmol/L), normomagnesemia (0.75–1.02 mmol/L), and hypermagnesemia (>1.02 mmol/L).

The primary outcome was HA-SBP. Community-acquired SBP was defined as SBP diagnosed at admission or within 48 hours after admission. HA-SBP was defined as SBP diagnosed more than 48 hours after admission (19, 20). SBP was diagnosed based on an ascitic fluid polymorphonuclear leukocyte count ≥ 0.25 × 10^9^/L (250 cells/mm³) and/or a positive ascitic fluid bacterial culture, regardless of the presence of clinical symptoms (21, 22). In clinical practice, diagnostic paracentesis or therapeutic paracentesis drainage is performed only when patients with cirrhosis have clinical indications. The secondary outcome was sepsis, identified using discharge diagnosis codes (ICD-10 codes A40.x and A41.x), with septic shock identified using ICD-10 code R57.2. Patients with sepsis at admission were excluded from the analysis of the sepsis outcome (N = 4).

### Data collection and management

We collected the following data from the hospital electronic medical record system: age, sex, hepatitis B virus infection, hepatitis C virus infection, alcohol use history, hypertension, diabetes mellitus, renal disease, severe liver disease, cancer, Charlson Comorbidity Index, mean arterial pressure, high-sensitivity C-reactive protein, white blood cell count, platelet count, serum albumin, total bilirubin, serum creatinine, serum sodium, serum magnesium, international normalized ratio, Model for End-Stage Liver Disease (MELD) 3.0 score, and diuretic use. Comorbidities were identified through review of medical records and discharge diagnosis codes. Mean arterial pressure and laboratory variables were defined as the first values measured within 48 hours of admission. The MELD 3.0 score was calculated preferentially using variables measured within 24 hours of admission; if these variables were unavailable, data obtained between 24 and 48 hours after admission were used. Diuretic use was defined as the use of diuretics between admission and serum magnesium measurement and was further categorized into loop diuretics and potassium-sparing diuretics.

In real-world studies, outliers and missing values are common. For laboratory variables, outliers were identified using the percentile method, and values below the 1st percentile or above the 99th percentile were considered outliers and treated as missing values. To reduce bias caused by missing data, variables with more than 30% missing values were excluded (**Supplementary Figure S1**). For variables included in the analysis, five imputation algorithms (mean, predictive mean matching, lasso.norm, random forest, and classification and regression tree) were used to generate five imputed datasets, and the mean of the imputed values was used as the final analytical dataset (23).

### Statistical analysis

All statistical analyses were performed using R software (version 4.5.0). Continuous variables with a normal or approximately normal distribution were presented as mean ± standard deviation, whereas non-normally distributed continuous variables were presented as median (interquartile range). Categorical variables were presented as counts and percentages. For between-group comparisons, normally distributed continuous variables were analyzed using one-way analysis of variance, non-normally distributed continuous variables were analyzed using the Kruskal–Wallis rank-sum test, and categorical variables were analyzed using the chi-square test or Fisher’s exact test.

Multivariable logistic regression models were used to evaluate the independent associations between serum magnesium levels and HA-SBP and sepsis, with results reported as odds ratios (ORs) and 95% confidence intervals (CIs). Potential confounders were selected based on previous literature, clinical relevance, and univariable analysis results, and variance inflation factors (VIFs) were used to assess multicollinearity among variables in the models. A VIF > 5 was considered indicative of multicollinearity (**Supplementary Figure S2**). Three models were constructed. Model 1 was an unadjusted model. Model 2 was adjusted for age, sex, diabetes mellitus, severe liver disease, and Charlson Comorbidity Index. Model 3 was further adjusted for mean arterial pressure, white blood cell count, high-sensitivity C-reactive protein, serum albumin, total bilirubin, serum sodium, international normalized ratio, MELD 3.0 score, loop diuretic use and potassium-sparing diuretic use in addition to the variables included in Model 2.

To explore the dose–response relationships between serum magnesium levels and the study outcomes, restricted cubic spline (RCS) regression analyses were performed with adjustment for the covariates included in Model 3. Subgroup analyses were conducted to assess the consistency of the associations across clinically relevant subgroups. Subgroup variables included age, sex, diabetes mellitus, severe liver disease, hepatitis B virus infection, and MELD 3.0 score, and interactions were evaluated using likelihood ratio tests. Receiver operating characteristic (ROC) curve analysis was performed to evaluate the predictive performance of serum magnesium levels for HA-SBP and sepsis.

We performed seven sensitivity analyses to assess the robustness of the results. First, we excluded patients who had fever at admission and leukocytosis within 48 hours after admission. Second, we excluded patients who had abdominal pain at admission and leukocytosis within 48 hours after admission. Third, we adjusted the thresholds for serum magnesium and categorized it into four groups. Fourth, we restricted the analysis to patients with ascites within 48 hours after admission. Fifth, we excluded patients who received magnesium supplementation within 48 hours after admission. Sixth, we excluded patients with diarrhea at admission. Seventh, we excluded patients who received sodium–glucose cotransporter 2 inhibitors within 48 hours after admission. All tests were two-sided, and P < 0.05 was considered statistically significant.

## Results

### Baseline characteristics

After application of the exclusion criteria, a total of 1110 hospitalized adult patients with cirrhosis were included. The detailed patient selection flowchart is shown in **Figure 1**. The mean age was 63.7 ± 11.9 years, and 64.44% were male. The median time from admission to SBP diagnosis in the HA-SBP group was 5.42 days (IQR, 3.69–8.77 days). The overall incidences of HA-SBP and sepsis were 12.25% (136/1110) and 6.06% (67/1106), respectively (**Table 1**).

**Figure 1:**
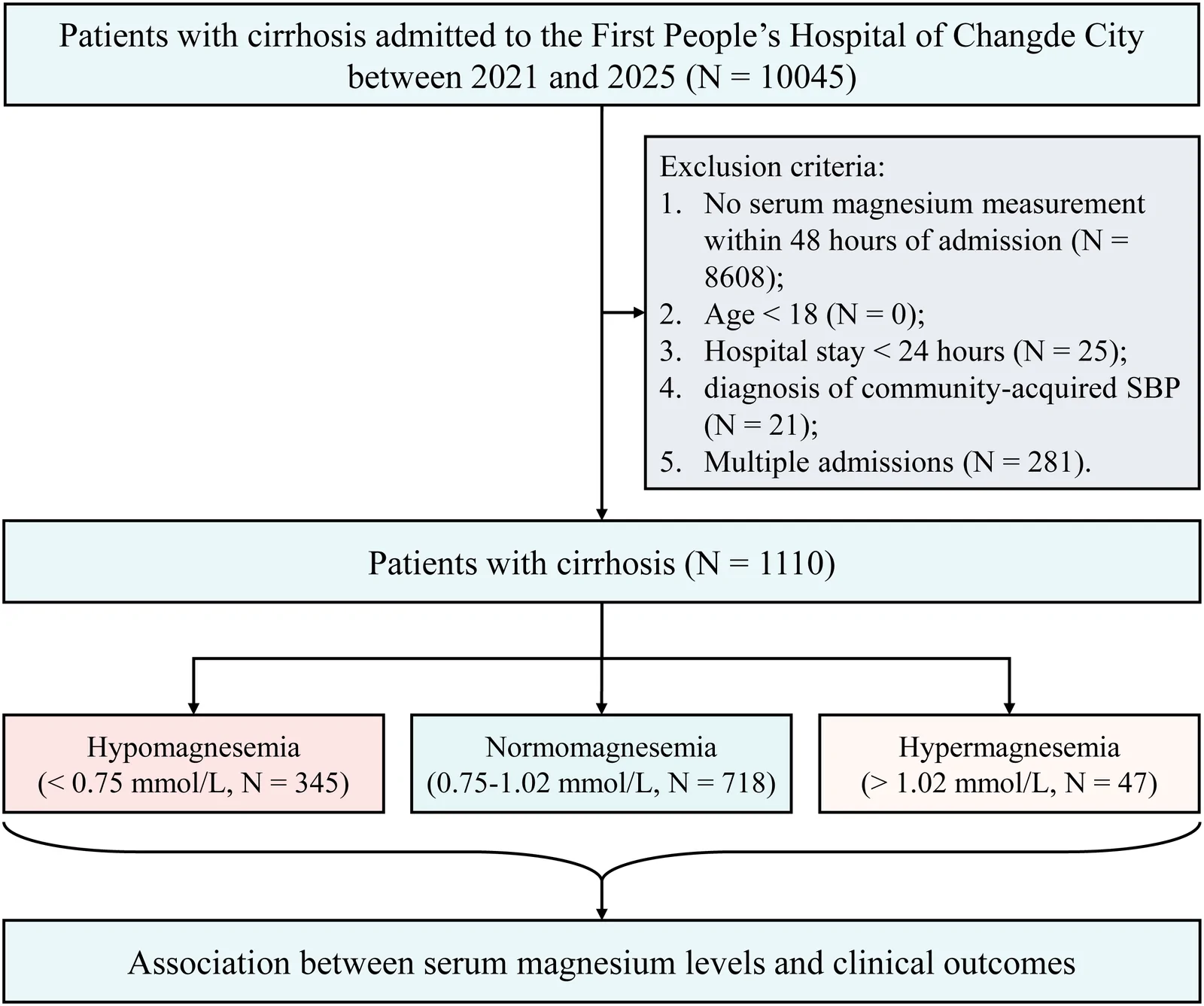
Study flow diagram of patients with cirrhosis included in the analysis. SBP, spontaneous bacterial peritonitis.

**Table 1:** Baseline clinical characteristics of patients with cirrhosis stratified by serum magnesium levels.

Characteristics	Overall (N = 1110)	Hypomagnesemia (< 0.75 mmol/L, N = 345)	Normomagnesemia (0.75 - 1.02 mmol/L, N = 718)	Hypermagnesemia (> 1.02 mmol/L, N = 47)	P*-*value
Magnesium (mmol/L)	0.80 (0.13)	0.66 (0.07)	0.85 (0.07)	1.12 (0.13)	<0.001
Age (yr)	63.7 (11.9)	63.0 (11.7)	63.9 (12.1)	64.7 (11.1)	0.400
Sex, n (%)					0.557
Female	395 (35.6%)	115 (33.3%)	262 (36.5%)	18 (38.3%)	
Male	715 (64.4%)	230 (66.7%)	456 (63.5%)	29 (61.7%)	
Medical history, n (%)					
Hepatitis B virus infection	372 (33.5%)	105 (30.4%)	252 (35.1%)	15 (31.9%)	0.312
Hepatitis C virus infection	57 (5.1%)	24 (7.0%)	31 (4.3%)	2 (4.3%)	0.180
Alcohol use	192 (17.3%)	76 (22.0%)	109 (15.2%)	7 (14.9%)	0.020
Hypertension	406 (36.6%)	113 (32.8%)	275 (38.3%)	18 (38.3%)	0.207
Diabetes mellitus	400 (36.0%)	130 (37.7%)	260 (36.2%)	10 (21.3%)	0.088
Renal disease	272 (24.5%)	85 (24.6%)	173 (24.1%)	14 (29.8%)	0.678
Severe liver disease	505 (45.5%)	184 (53.3%)	292 (40.7%)	29 (61.7%)	<0.001
Cancer	185 (16.7%)	51 (14.8%)	121 (16.9%)	13 (27.7%)	0.083
Charlson Comorbidity Index	6.0 (4.0, 8.0)	6.0 (4.0, 8.0)	6.0 (4.0, 8.0)	7.0 (5.0, 9.0)	0.101
Mean arterial pressure, mmHg	91.0 (14.1)	88.4 (15.7)	91.9 (13.1)	95.6 (13.1)	<0.001
High-sensitivity CRP, mg/L	12.1 (3.4, 31.2)	15.0 (5.6, 39.8)	10.9 (2.8, 27.2)	6.7 (1.8, 26.2)	<0.001
White blood cell count, ×10^9^/L	5.3 (3.8, 7.4)	5.6 (3.8, 8.8)	5.1 (3.8, 7.0)	5.6 (3.6, 8.1)	0.003
Platelet count, ×10^9^/L	96.8 (62.0, 141.0)	87.0 (57.0, 126.0)	102.0 (65.0, 145.7)	94.0 (56.0, 135.0)	0.006
Serum albumin, g/L	33.2 (6.6)	30.4 (6.5)	34.4 (6.1)	36.5 (7.3)	<0.001
Total bilirubin, μmol/L	18.8 (11.2, 31.5)	21.6 (11.8, 40.3)	16.9 (10.7, 28.0)	23.8 (12.5, 38.5)	<0.001
Serum creatinine, μmol/L	78.0 (60.0, 121.0)	77.0 (58.0, 119.0)	78.0 (60.0, 122.0)	76.0 (61.0, 122.5)	0.631
Serum sodium, mmol/L	138.8 (3.9)	138.2 (4.2)	139.2 (3.7)	138.1 (4.1)	<0.001
International normalized ratio	1.2 (0.3)	1.3 (0.3)	1.2 (0.3)	1.2 (0.4)	<0.001
MELD 3.0 score	11.2 (7.3, 18.2)	14.5 (8.9, 20.4)	9.7 (7.3, 16.3)	9.5 (7.3, 18.2)	<0.001
Diuretic use, n (%)	208 (18.7%)	74 (21.4%)	124 (17.3%)	10 (21.3%)	0.237
Loop diuretic use	182 (16.4%)	61 (17.7%)	112 (15.6%)	9 (19.1%)	0.604
Potassium-sparing diuretic use	71 (6.4%)	21 (6.1%)	41 (5.7%)	9 (19.1%)	0.005
Time to HA-SBP diagnosis, days	5.4 (3.7, 8.8)	6.1 (5.2, 12.7)	4.4 (3.5, 7.5)	6.9 (4.1, 8.0)	0.290
Length of hospital stay, days	7.9 (5.6, 12.0)	8.8 (5.9, 13.1)	7.6 (5.1, 11.2)	7.7 (5.2, 11.5)	0.007
HA-SBP, n (%)	136 (12.25%)	64 (18.55%)	65 (9.05%)	7 (14.89%)	<0.001
Sepsis, n (%)	67 (6.06%)	39 (11.34%)	27 (3.78%)	1 (2.13%)	<0.001

Continuous data are expressed as mean (SD) or median (IQR).

CRP, C-reactive protein; MELD, Model for End-Stage Liver Disease; HA-SBP, hospital-acquired spontaneous bacterial peritonitis; SD, standard deviation; IQR, interquartile range.

**Supplementary Figure S3** shows the distribution of serum magnesium levels, with a mean value of 0.80 ± 0.13 mmol/L. Compared with patients without HA-SBP, those with HA-SBP had significantly lower serum magnesium levels (0.75 ± 0.14 mmol/L vs 0.81 ± 0.13 mmol/L, P < 0.001). Similarly, patients with sepsis had significantly lower serum magnesium levels than those without sepsis (0.72 ± 0.16 mmol/L vs 0.81 ± 0.13 mmol/L, P < 0.001) (**Figure 2**).

**Figure 2:**
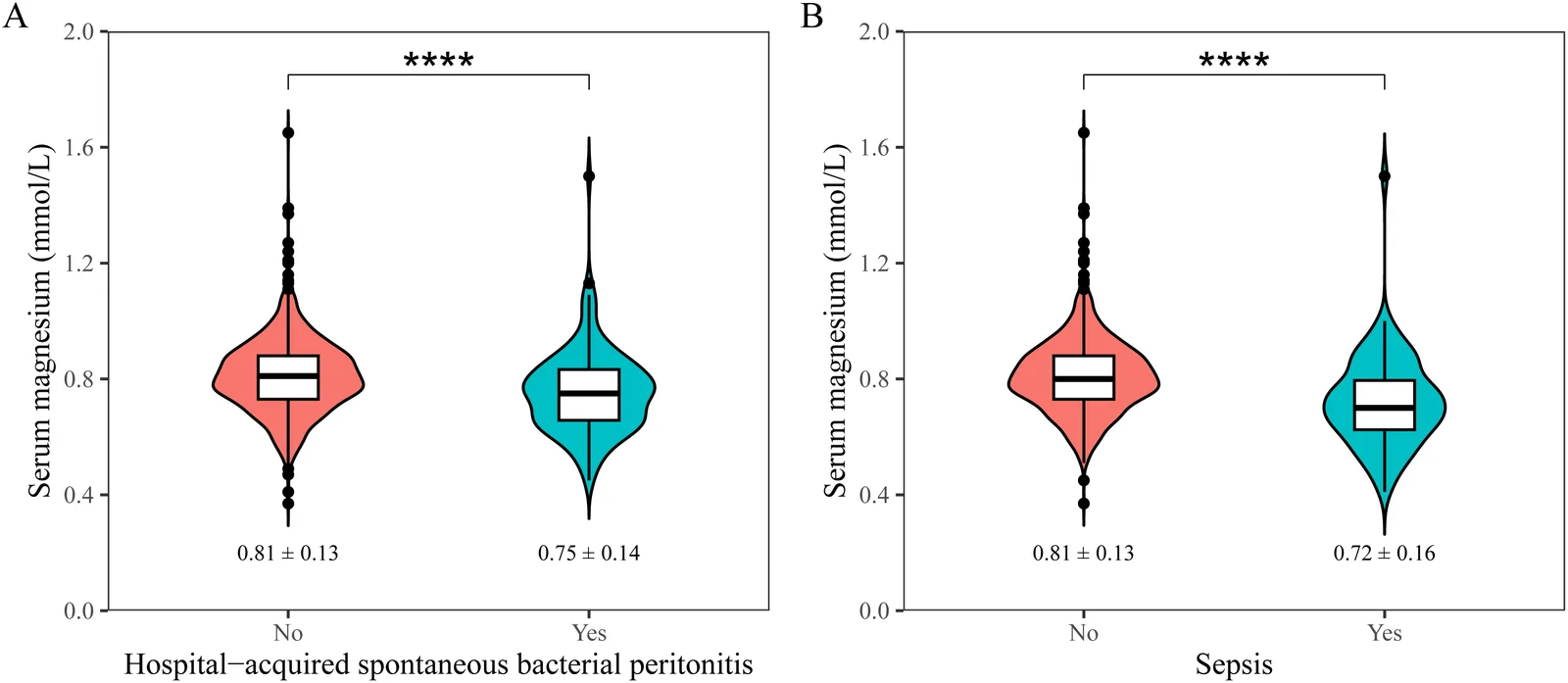
Serum magnesium levels stratified by outcome status. **(A)** Hospital-acquired spontaneous bacterial peritonitis. **(B)** Sepsis. **** indicates P < 0.0001.

Baseline characteristics according to serum magnesium status are shown in **Table 1**. The overall prevalence of hypomagnesemia was 31.08% (345/1110). Compared with normomagnesemia, the hypomagnesemia group had higher proportions of alcohol use and severe liver disease. In addition, the hypomagnesemia group had lower mean arterial pressure, platelet count, serum albumin, and serum sodium, but higher high-sensitivity C-reactive protein, white blood cell count, total bilirubin, international normalized ratio, MELD 3.0 score, and a longer hospital stay (**Table 1**).

Furthermore, the incidences of HA-SBP were 18.55%, 9.05%, and 14.89% in the hypomagnesemia, normomagnesemia, and hypermagnesemia groups, respectively (P < 0.001), whereas the corresponding incidences of sepsis were 11.34%, 3.78%, and 2.13%, respectively (P < 0.001) (**Table 1**).

### Univariable logistic regression analysis for HA-SBP

The results of the univariable logistic regression analysis are shown in **Table 2**. In hospitalized patients with cirrhosis, serum magnesium levels (P < 0.001), diabetes mellitus (P = 0.023), severe liver disease (P < 0.001), Charlson Comorbidity Index (P = 0.011), mean arterial pressure (P = 0.007), white blood cell count (P < 0.001), high-sensitivity C-reactive protein (P < 0.001), serum albumin (P < 0.001), total bilirubin (P < 0.001), serum sodium (P < 0.001), international normalized ratio (P < 0.001), MELD 3.0 score (P < 0.001), and loop diuretic use (P = 0.033) were all significantly associated with HA-SBP.

**Table 2:** Univariable logistic regression for hospital-acquired spontaneous bacterial peritonitis in patients with cirrhosis.

Characteristics	OR (95% CI)	P-value
Magnesium as continuous variable (mmol/L)	0.029 (0.006, 0.128)	<0.001
Magnesium as categorical variable		
Normomagnesemia (0.75-1.02 mmol/L)	Ref	
Hypomagnesemia (< 0.75 mmol/L)	2.288 (1.576, 3.323)	<0.001
Hypermagnesemia (> 1.02 mmol/L)	1.758 (0.698, 3.857)	0.189
Age (yr)	0.991 (0.976, 1.006)	0.213
Sex, n (%)		
Female	Ref	
Male	1.224 (0.838, 1.812)	0.303
Medical history, n (%)		
Hepatitis B virus infection	1.016 (0.691, 1.477)	0.935
Hepatitis C virus infection	0.677 (0.232, 1.571)	0.413
Alcohol use	1.150 (0.714, 1.795)	0.549
Hypertension	0.720 (0.484, 1.055)	0.098
Diabetes mellitus	0.629 (0.417, 0.930)	0.023
Renal disease	1.388 (0.927, 2.047)	0.104
Severe liver disease	2.950 (2.024, 4.363)	<0.001
Cancer	1.349 (0.847, 2.088)	0.192
Charlson Comorbidity Index	1.096 (1.020, 1.177)	0.011
Mean arterial pressure, mmHg	0.982 (0.970, 0.995)	0.007
High-sensitivity C-reactive protein, mg/L	1.009 (1.005, 1.013)	<0.001
White blood cell count, ×10^9^/L	1.120 (1.078, 1.164)	<0.001
Platelet count, ×10^9^/L	0.999 (0.996, 1.002)	0.383
Serum albumin, g/L	0.902 (0.874, 0.930)	<0.001
Total bilirubin, μmol/L	1.009 (1.005, 1.012)	<0.001
Serum creatinine, μmol/L	1.000 (0.999, 1.001)	0.924
Serum sodium, mmol/L	0.900 (0.862, 0.940)	<0.001
International normalized ratio	4.728 (2.945, 7.597)	<0.001
MELD 3.0 score	1.081 (1.059, 1.104)	<0.001
Diuretic use, n (%)	1.395 (0.897, 2.120)	0.128
Loop diuretic use	1.609 (1.026, 2.464)	0.033
Potassium-sparing diuretic use	0.904 (0.392, 1.824)	0.794

MELD, Model for End-Stage Liver Disease; OR, odds ratio; CI, confidence interval.

### Association of serum magnesium levels with the risks of HA-SBP and sepsis

In the multivariable logistic regression analysis, when serum magnesium was analyzed as a continuous variable, higher serum magnesium levels were significantly associated with lower risks of HA-SBP and sepsis. After further adjustment for confounders in Model 3, per 1-SD increase in serum magnesium was associated with a 20.8% reduction in the risk of HA-SBP (OR = 0.792, 95% CI: 0.642–0.970, P = 0.026) and a 35.5% reduction in the risk of sepsis (OR = 0.645, 95% CI: 0.464–0.883, P = 0.008) (**Table 3**).

**Table 3:** Association of serum magnesium levels with HA-SBP and sepsis in patients with cirrhosis. .

Characteristics	Event rate, n / N (%)	Model 1		Model 2		Model 3	
		OR (95% CI)	P-value	OR (95% CI)	P-value	OR (95% CI)	P-value
**HA-SBP**	136 / 1110 (12.25%)						
Magnesium as continuous (per 1-SD increase)		0.629 (0.516, 0.764)	<0.001	0.653 (0.535, 0.792)	<0.001	0.792 (0.642, 0.970)	0.026
Magnesium as categorical variable							
Normomagnesemia (0.75-1.02 mmol/L)	65 / 718 (9.05%)	Ref		Ref		Ref	
Hypomagnesemia (< 0.75 mmol/L)	64 / 345 (18.55%)	2.288 (1.576, 3.323)	<0.001	2.075 (1.415, 3.042)	<0.001	1.520 (1.009, 2.286)	0.044
Hypermagnesemia (> 1.02 mmol/L)	7 / 47 (14.89%)	1.758 (0.698, 3.857)	0.189	1.317 (0.514, 2.953)	0.531	1.470 (0.526, 3.611)	0.428
P for trend			0.002		0.004		0.193
**Sepsis**	67 / 1106 (6.06%)						
Magnesium as continuous (per 1-SD increase)		0.474 (0.358, 0.624)	<0.001	0.476 (0.357, 0.629)	<0.001	0.645 (0.464, 0.883)	0.008
Magnesium as categorical variable							
Normomagnesemia (0.75-1.02 mmol/L)	27 / 715 (3.78%)	Ref		Ref		Ref	
Hypomagnesemia (< 0.75 mmol/L)	39 / 344 (11.34%)	3.258 (1.968, 5.470)	<0.001	3.113 (1.870, 5.251)	<0.001	2.213 (1.189, 4.177)	0.013
Hypermagnesemia (> 1.02 mmol/L)	1 / 47 (2.13%)	0.554 (0.031, 2.694)	0.566	0.498 (0.028, 2.444)	0.499	0.586 (0.028, 3.852)	0.642
P for trend			<0.001		<0.001		0.011

Model 1: unadjusted.

Model 2: adjusted for age, sex, diabetes mellitus, severe liver disease, Charlson Comorbidity Index.

Model 3: adjusted for age, sex, diabetes mellitus, severe liver disease, Charlson Comorbidity Index, mean arterial pressure, white blood cell count, high-sensitivity C-reactive protein, serum albumin, total bilirubin, serum sodium, international normalized ratio, MELD 3.0 score, loop diuretic use, and potassium-sparing diuretic use.

HA-SBP, hospital-acquired spontaneous bacterial peritonitis; MELD, Model for End-Stage Liver Disease; SD, standard deviation; OR, odds ratio; CI, confidence interval.

When serum magnesium was analyzed as a categorical variable, compared with normomagnesemia, hypomagnesemia had significantly higher risks of HA-SBP (Model 3: OR = 1.520, 95% CI: 1.009–2.286, P = 0.044) and sepsis (Model 3: OR = 2.213, 95% CI: 1.189–4.177, P = 0.013). In contrast, hypermagnesemia was not significantly associated with HA-SBP or sepsis (all P > 0.05) (**Table 3**). The results of the sensitivity analyses were consistent with those of the main analysis (**Supplementary Table S1**).

The adjusted RCS curves showed negative associations between serum magnesium levels and both HA-SBP and sepsis (**Figure 3**). No significant nonlinear relationship was observed for either outcome (both P for nonlinearity > 0.05), suggesting an overall linear trend. As serum magnesium levels increased, the risks of HA-SBP and sepsis gradually decreased. The decline was steeper within the range of 0.50–0.75 mmol/L, whereas the reduction was relatively limited within the range of 0.75–1.10 mmol/L (**Figure 3**). Further sensitivity analysis showed that, compared with the [0.75, 1.02] mmol/L group, serum magnesium <0.65 mmol/L was associated with an increased risk of HA-SBP (OR = 1.851, 95% CI: 1.052–3.202, P = 0.030) and sepsis (OR = 3.830, 95% CI: 1.730–8.501, P < 0.001). In contrast, the [0.65, 0.75) mmol/L group was not significantly associated with the risk of HA-SBP or sepsis (**Supplementary Table S1**).

**Figure 3:**
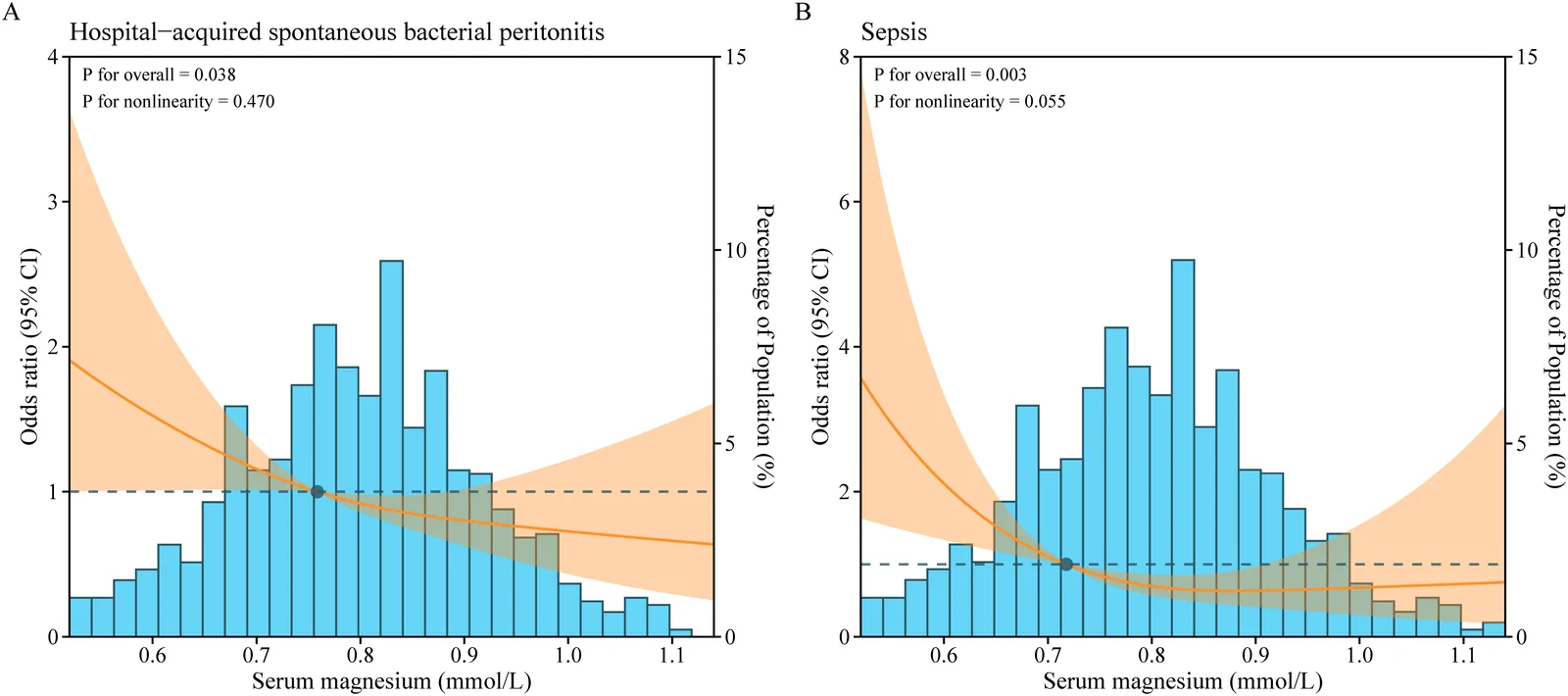
Association between serum magnesium levels and outcomes in patients with cirrhosis. **(A)** Hospital-acquired spontaneous bacterial peritonitis. **(B)** Sepsis. CI, confidence interval.

### Subgroup analysis

We further performed subgroup analyses stratified by age, sex, diabetes mellitus, severe liver disease, hepatitis B virus infection, and MELD 3.0 score. The results showed that the associations between serum magnesium and HA-SBP were generally consistent across subgroups (all P values for interaction > 0.05, **Supplementary Figure S4**).

### Predictive performance of serum magnesium levels

We evaluated the predictive performance of serum magnesium levels for HA-SBP and sepsis (**Supplementary Figure S5**). The results showed that the areas under the receiver operating characteristic curves (AUCs) of serum magnesium for predicting HA-SBP and sepsis were 0.635 (95% CI: 0.583–0.686) and 0.688 (95% CI: 0.617–0.758), respectively, suggesting modest discriminatory ability.

## Discussion

### Main finding

This real-world retrospective cohort study evaluated the association between the first serum magnesium measurement obtained within 48 hours of admission and the risks of HA-SBP and sepsis in hospitalized patients with cirrhosis. The results showed that lower serum magnesium levels at admission were significantly associated with increased risks of HA-SBP and sepsis. RCS analysis further demonstrated an overall linear inverse association between serum magnesium levels and the risks of HA-SBP and sepsis. This association was generally consistent across different subgroups. Sensitivity analyses yielded consistent results. These findings suggest that serum magnesium may be a potential biomarker for identifying patients with cirrhosis at high risk of infectious complications.

### Relation with previous evidence

Previous studies have shown that the prevalence of magnesium deficiency or hypomagnesemia in hospitalized patients with cirrhosis is approximately 34%–60% (8, 24). In the present study, the prevalence of hypomagnesemia was 31% among hospitalized patients with cirrhosis, which was comparable to previous reports, suggesting that low magnesium status is common in this population. Meanwhile, the incidence of HA-SBP in our study was 12.25%, which was generally consistent with the reported pooled prevalence of 14.08% in the Asia-Pacific region (2), indicating a substantial infectious burden in hospitalized patients with cirrhosis. This study found that hypomagnesemia was significantly associated with increased risks of HA-SBP and sepsis. As serum magnesium levels increased, the risks of HA-SBP and sepsis gradually decreased, with a more pronounced decline within the range of 0.50–0.75 mmol/L, particularly 0.50–0.65 mmol/L, and a less pronounced decline within the range of 0.75–1.10 mmol/L. Although previous evidence has mainly been derived from kidney transplant recipients and patients with systemic inflammatory response syndrome, it generally supports an association between low magnesium status and increased susceptibility to infection (13–15). In a study of 376 kidney transplant recipients, after adjustment for confounding factors, magnesium deficiency was an independent predictor of urinary tract infection and viral infection (14). Another study of 873 kidney transplant recipients showed that low serum magnesium was significantly associated with increased infection risk, and among patients with baseline serum magnesium <2 mg/dL, each 0.1 mg/dL decrease in serum magnesium was associated with a 15% increase in infection risk (13). In addition, among 2589 patients with systemic inflammatory response syndrome, hypomagnesemia (<1.5 mg/dL) remained significantly associated with an increased risk of septic shock after adjustment for potential confounders (15). These findings suggest that low magnesium may promote the development and progression of infection by affecting host immune function (7). Compared with previous studies, the present study focused on infection-related complications specific to patients with cirrhosis and, for the first time, simultaneously evaluated the associations of hypomagnesemia with HA-SBP and sepsis, thereby expanding the clinical significance of serum magnesium in this population. This study further showed that serum magnesium levels had modest predictive value for HA-SBP and sepsis (AUC 0.635–0.688), suggesting that serum magnesium may serve as a potential biomarker for early identification of patients at high risk of infection. Although its predictive performance was limited, serum magnesium warrants further investigation for clinical use in infection risk stratification among patients with cirrhosis because of its simple measurement and low cost.

### Possible explanations for the findings

The mechanisms underlying the increased risks of HA-SBP and sepsis associated with hypomagnesemia may mainly involve immune dysfunction, exaggerated inflammation, and increased oxidative stress (25). Magnesium participates in regulating the function of natural killer cells and T cells, and low magnesium status may impair host antimicrobial defense (7). At the same time, hypomagnesemia may promote the activation of macrophages and leukocytes, increase the release of proinflammatory cytokines such as tumor necrosis factor-α, interleukin-1β, and interleukin-6, and further amplify inflammatory responses and oxidative stress through increased intracellular Ca²^+^ and activation of inflammatory pathways such as nuclear factor kappa B (26). In patients with cirrhosis, factors such as hypoalbuminemia, secondary hyperaldosteronism, and abnormal transient receptor potential melastatin 7 expressions may make hypomagnesemia more likely to persist, while impaired liver function and immune imbalance further reduce the ability to clear bacterial translocation and intra-abdominal infection, thereby increasing the risk of HA-SBP (7, 27, 28). Once infection occurs, the hypomagnesemia-related inflammatory cascade may also promote the systemic spread of infection, ultimately increasing the risk of sepsis (15).

### Implications for clinical practice

The findings of this study have potential clinical implications. First, serum magnesium is a routine and readily available laboratory marker that may be used for early identification of infection risk in patients with cirrhosis because of its low cost and ease of measurement. For patients with hypomagnesemia at admission, clinicians may intensify monitoring for HA-SBP and sepsis, thereby enabling earlier intervention. Second, magnesium levels are potentially modifiable. Previous studies have suggested that appropriate magnesium supplementation may improve immune function and inflammatory status; therefore, serum magnesium may represent a potential therapeutic target (29–31). A real-world study showed that magnesium sulfate supplementation during an intensive care unit stay was associated with lower in-hospital mortality and 180-day mortality in patients with cirrhosis (32). However, the predictive performance of serum magnesium for HA-SBP and sepsis was relatively limited in the present study, suggesting that serum magnesium may be more suitable as an adjunctive marker for risk stratification rather than as a standalone clinical prediction tool (33). Future studies may consider combining serum magnesium with other inflammatory markers or scoring systems to improve predictive performance.

### Study limitations

This study also has several limitations. First, this was a single-center retrospective cohort study, which may be subject to selection bias. In addition, the etiological profile of cirrhosis and dietary habits in the Chinese population may have regional specificity, potentially limiting the generalizability of our findings. Therefore, multicenter prospective studies are still warranted for further validation. Second, although multiple potential confounders were adjusted for in the multivariable models, residual confounding from factors such as Child–Pugh class, history of previous SBP, upper gastrointestinal bleeding, pre-admission magnesium supplementation, pre-admission long-term proton pump inhibitor use, pre-admission long-term sodium–glucose cotransporter 2 inhibitor use, antibiotic treatment, and nutritional status cannot be completely excluded. Third, only the first serum magnesium level measured within 48 hours after admission was analyzed, and changes in magnesium levels during hospitalization were not assessed over time; therefore, the influence of temporal changes during disease progression on outcomes could not be evaluated. In addition, the potential therapeutic effect of magnesium supplementation on HA-SBP or sepsis was not evaluated. Fourth, serum magnesium reflects only extracellular magnesium levels and cannot fully represent total body magnesium or intracellular magnesium status, which may underestimate its physiological role (4). Fifth, due to incomplete data, the study could not completely exclude the possibility that some patients already had SBP and sepsis at admission or that delayed diagnosis occurred in some cases. This may have led to overestimation of the incidence of HA-SBP and sepsis. However, the results of multiple sensitivity analyses were consistent with the main analysis, supporting the robustness of our findings. Sixth, because paracentesis was not routinely performed in all patients but was conducted based on clinical indications, the possibility of delayed sampling or misclassification cannot be completely excluded. Sepsis ascertainment based on diagnostic codes has high specificity but may miss patients meeting Sepsis-3 criteria, potentially underestimating the incidence of sepsis. Finally, because of the observational study design, a causal relationship between serum magnesium levels and HA-SBP or sepsis cannot be established.

## Conclusions

Hypomagnesemia was associated with increased risks of HA-SBP and sepsis in patients with cirrhosis, and these findings should be further validated in prospective studies.

## Data Availability

The data analyzed in this study is subject to the following licenses/restrictions: The data that support the findings of this study are available from the corresponding author upon reasonable request. Requests to access these datasets should be directed to Wan-Jie Gu(guwanjie@jnu.edu.cn).

## References

[1] Piano S Bunchorntavakul C Marciano S Reddy KR . Infections in cirrhosis. Lancet Gastroenterol Hepatol. (2024) 9:745–57. doi: 10.1016/S2468-1253(24)00078-5 38754453

[2] Tay PWL Xiao JL Tan DJH Ng C Lye YN Lim WH . An epidemiological meta-analysis on the worldwide prevalence, resistance, and outcomes of spontaneous bacterial peritonitis in cirrhosis. Front Med (Lausanne). (2021) 8:693652. doi: 10.3389/fmed.2021.693652 34422858 PMC8375592

[3] Schmidt ML Barritt AS Orman ES Hayashi PH . Decreasing mortality among patients hospitalized with cirrhosis in the United States from 2002 through 2010. Gastroenterology. (2015) 148:967–977.e2. doi: 10.1053/j.gastro.2015.01.032 25623044 PMC4430328

[4] Fawcett WJ Haxby EJ Male DA . Magnesium: physiology and pharmacology. Br J Anaesth. (1999) 83:302–20. doi: 10.1093/bja/83.2.302 10618948

[5] Arnaud MJ . Update on the assessment of magnesium status. Br J Nutr. (2008) 99:S24–36. doi: 10.1017/S000711450800682X 18598586

[6] Papagiannidou A Mitropoulou M Papantzikos K Petropoulou D Tsilingiris D Magkos F . Hypomagnesemia: a clinical and nutritional update. Curr Nutr Rep. (2026) 15:30. doi: 10.1007/s13668-026-00745-5 41872423 PMC13009017

[7] Yang ZY Li J Zhang J Sun C . Magnesium deficiency: the insidious executor of the liver disease. J Am Nutr Assoc. (2025) 44:439–53. doi: 10.1080/27697061.2024.2443063 39718301

[8] Peng X Xiang RY Li XF Tian H Li CQ Peng Z . Magnesium deficiency in liver cirrhosis: a retrospective study. Scand J Gastroenterol. (2021) 56:463–8. doi: 10.1080/00365521.2021.1888154 33646920

[9] Parisse S Gianoncelli A Isani G Gambaro FL Andreani G Malucelli E . Severity of hepatocyte damage and prognosis in cirrhotic patients correlate with hepatocyte magnesium depletion. Nutrients. (2023) 15:2626. doi: 10.3390/nu15112626 37299589 PMC10255194

[10] Lin BW Xiao WB Huang P Lin XX Lin YX Lin JD . Association between serum magnesium concentrations and the risk of developing acute kidney injury in patients with cirrhosis: a retrospective cohort study based on the MIMIC-IV database. Ren Fail. (2024) 46:2368088. doi: 10.1080/0886022X.2024.2368088 39108151 PMC11308976

[11] Bravo M Simón J González-Recio I Martinez-Cruz LA Goikoetxea-Usandizaga N Martínez-Chantar ML . Magnesium and liver metabolism through the lifespan. Adv Nutr. (2023) 14:739–51. doi: 10.1016/j.advnut.2023.05.009 37207838 PMC10334155

[12] Cohen-Hagai K Feldman D Turani-Feldman T Hadary R Lotan S Kitay-Cohen Y . Magnesium deficiency and minimal hepatic encephalopathy among patients with compensated liver cirrhosis. Isr Med Assoc J. (2018) 20:533–8. Available online at: https://pubmed.ncbi.nlm.nih.gov/30221864/. 30221864

[13] Odler B Deak AT Pregartner G Riedl R Bozic J Trummer C . Hypomagnesemia is a risk factor for infections after kidney transplantation: a retrospective cohort analysis. Nutrients. (2021) 13:1296. doi: 10.3390/nu13041296 33919913 PMC8070921

[14] Van Laecke S Vermeiren P Nagler EV Caluwe R De Wilde M Van der Vennet M . Magnesium and infection risk after kidney transplantation: an observational cohort study. J Infect. (2016) 73:8–17. doi: 10.1016/j.jinf.2016.04.007 27084308

[15] Thongprayoon C Cheungpasitporn W Erickson SB . Admission hypomagnesemia linked to septic shock in patients with systemic inflammatory response syndrome. Ren Fail. (2015) 37:1518–21. doi: 10.3109/0886022X.2015.1074519 26335852

[16] Garrido-Trevino L Ahn S Tapper EB Walker OC Ogola G Asrani SK . Diagnosis of acute-on-chronic liver failure in critically ill patients with cirrhosis using administrative data. Am J Gastroenterol. (2025) 120:2443–7. doi: 10.14309/ajg.0000000000003422 40094305

[17] Mapakshi S Kramer JR Richardson P El-Serag HB Kanwal F . Positive predictive value of international classification of diseases, 10th revision, codes for cirrhosis and its related complications. Clin Gastroenterol Hepatol. (2018) 16:1677–8. doi: 10.1016/j.cgh.2018.01.042 29410051

[18] von Elm E Altman DG Egger M Pocock SJ Gøtzsche PC Vandenbroucke JP . The strengthening the reporting of observational studies in epidemiology (STROBE) statement: guidelines for reporting observational studies. PloS Med. (2007) 4:e296. doi: 10.1371/journal.pmed.0040296 17941714 PMC2020495

[19] Shi L Wu D Wei L Liu SX Zhao P Tu B . Nosocomial and community-acquired spontaneous bacterial peritonitis in patients with liver cirrhosis in China: comparative microbiology and therapeutic implications. Sci Rep. (2017) 7:46025. doi: 10.1038/srep46025 28382951 PMC5382543

[20] Bert F Andreu M Durand F Degos F Galdbart JO Moreau R . Nosocomial and community-acquired spontaneous bacterial peritonitis: comparative microbiology and therapeutic implications. Eur J Clin Microbiol Infect Dis. (2003) 22:10–5. doi: 10.1007/s10096-002-0840-z 12582738

[21] Runyon BA . Introduction to the revised American Association for the Study of Liver Diseases Practice Guideline management of adult patients with ascites due to cirrhosis 2012. Hepatology. (2013) 57:1651–3. doi: 10.1002/hep.26359 23463403

[22] European Association for the Study of the Liver . EASL clinical practice guidelines on the management of ascites, spontaneous bacterial peritonitis, and hepatorenal syndrome in cirrhosis. J Hepatol. (2010) 53:397–417. doi: 10.1016/j.jhep.2010.05.004 20633946

[23] Wang ZC Wang W Xu JY He Q Sun C Xie SY . Development and validation of dynamic clinical subphenotypes in acute pancreatitis patients using vital sign trajectories in intensive care units: a multinational cohort study. Signal Transduct Target Ther. (2025) 10:180. doi: 10.1038/s41392-025-02261-4 40467599 PMC12137743

[24] SafariKish B Rafiei F Ebrahimi A Azizi-Soleiman F Rafiei R . The relationship between plasma magnesium concentration and hepatic encephalopathy in liver cirrhosis patients: a preliminary result of a referral center in Iran. Middle East J Rehabil Health Stud. (2022) 9:e122978. doi: 10.5812/mejrh-122978

[25] Nielsen FH . Magnesium deficiency and increased inflammation: current perspectives. J Inflammation Res. (2018) 11:25–34. doi: 10.2147/JIR.S136742 29403302 PMC5783146

[26] Gu WJ Chen JL Duan XJ Zhang CY Huang Y Zhu MG . Magnesium sulfate alleviates sepsis-associated acute kidney injury by reducing inflammation, oxidative stress, and apoptosis via inhibiting the NF-κB pathway. FASEB J. (2026) 40:e71456. doi: 10.1096/fj.202502733R 41537746

[27] Ji LL Yu HH Wang RW Yan HM Yin XF Guo SS . Targeting magnesium homeostasis: a novel therapeutic strategy for liver diseases. Front Nutr. (2026) 13:1709477. doi: 10.3389/fnut.2026.1709477 41859661 PMC12995791

[28] Cohen MI McNamara H Finberg L . Serum magnesium in children with cirrhosis. J Pediatr. (1970) 76:453–5. doi: 10.1016/S0022-3476(70)80489-9 5413668

[29] Gu WJ Duan XJ Liu XZ Cen Y Tao LY Lyu J . Association of magnesium sulfate use with mortality in critically ill patients with sepsis: a retrospective propensity score-matched cohort study. Br J Anaesth. (2023) 131:861–70. doi: 10.1016/j.bja.2023.08.005 37684164

[30] Yin Y Costello RB Fonarow GC Heidenreich PA Morgan CJ Faselis C . Oral magnesium and outcomes in US veterans with heart failure. Eur Heart J. (2026) 47:80–90. doi: 10.1093/eurheartj/ehaf881 41338273 PMC12765560

[31] Barbosa EB Tomasi CD De Castro Damasio D Vinhas M Lichtenfels B de Luca Francisco V . Effects of magnesium supplementation on the incidence of acute kidney injury in critically ill patients presenting with hypomagnesemia. Intensive Care Med. (2016) 42:1084–5. doi: 10.1007/s00134-016-4276-9 26928037

[32] Chen BX Yang YP Liang MJ Kou YQ Ye RY Zhan LP . The use of magnesium sulfate can reduce the mortality risk of cirrhosis patients: a retrospective cohort study. Front Pharmacol. (2025) 16:1551495. doi: 10.3389/fphar.2025.1551495 41190021 PMC12580101

[33] Cheng X Wang W Xue YN Tang HL Li D . Serum magnesium predictive value in hepatocellular carcinoma patients on first-line immunotherapy: a retrospective study. Front Immunol. (2026) 16:1732557. doi: 10.3389/fimmu.2025.1732557 41562087 PMC12813027

