# Supplementary material for: Association of serum magnesium levels with the risk of spontaneous bacterial peritonitis and sepsis in hospitalized patients with cirrhosis: a retrospective cohort study
Source: Front Immunol. 2026 Sep 15;17:1857975. doi: 10.3389/fimmu.2026.1857975 (PMC13619515; doi:10.3389/fimmu.2026.1857975)
Supplement: Supplementary file 1 [file DataSheet1.docx]

**Supplementary Materials and Methods**

**A. Study population**

Cirrhosis was diagnosed based on International Classification of Diseases (ICD) codes (ICD-10: K702, K703, K7030, K7031, K717, K74, K741, K742, K743, K744, K745, K746, K7460, K7469).

| **ICD code** | **ICD version** | **Long title** |
| --- | --- | --- |
| K702 | 10 | Alcoholic fibrosis and sclerosis of liver |
| K703 | 10 | Alcoholic cirrhosis of liver |
| K7030 | 10 | Alcoholic cirrhosis of liver without ascites |
| K7031 | 10 | Alcoholic cirrhosis of liver with ascites |
| K717 | 10 | Toxic liver disease with fibrosis and cirrhosis of liver |
| K74 | 10 | Fibrosis and cirrhosis of liver |
| K741 | 10 | Hepatic sclerosis |
| K742 | 10 | Hepatic fibrosis with hepatic sclerosis |
| K743 | 10 | Primary biliary cirrhosis |
| K744 | 10 | Secondary biliary cirrhosis |
| K745 | 10 | Biliary cirrhosis, unspecified |
| K746 | 10 | Other and unspecified cirrhosis of liver |
| K7460 | 10 | Unspecified cirrhosis of liver |
| K7469 | 10 | Other cirrhosis of liver |

**Table S1. Sensitivity analysis of the association between serum magnesium levels and the risks of HA-SBP and sepsis in patients with cirrhosis.**

| **Sensitivity analysis** | **Characteristics** | **HA-SBP** | | | **Sepsis** | | |
| --- | --- | --- | --- | --- | --- | --- | --- |
|  |  | **Event rate,**  **n / N (%)** | **OR (95% CI)** * | **P-value** | **Event rate,**  **n / N (%)** | **OR (95% CI)** * | **P-value** |
| Sensitivity analysis 1 | Magnesium as continuous (per 1-SD increase) |  | 0.790 (0.636, 0.975) | 0.031 |  | 0.667 (0.463, 0.945) | 0.026 |
|  | Magnesium as categorical variable | 125 / 1071 (11.67%) |  |  | 51 / 1070 (4.77%) |  |  |
|  | Normomagnesemia (0.75-1.02 mmol/L) | 59 / 695 (8.49%) | Ref |  | 21 / 695 (3.02%) | Ref |  |
|  | Hypomagnesemia (< 0.75 mmol/L) | 60 / 331 (18.13%) | 1.535 (1.002, 2.345) | 0.048 | 29 / 330 (8.79%) | 2.039 (1.035, 4.069) | 0.040 |
|  | Hypermagnesemia (> 1.02 mmol/L) | 6 / 45 (13.33%) | 1.250 (0.424, 3.147) | 0.659 | 1 / 45 (2.22%) | 0.773 (0.038, 4.839) | 0.820 |
|  | P for trend |  |  | 0.143 |  |  | 0.047 |
| Sensitivity analysis 2 | Magnesium as continuous (per 1-SD increase) |  | 0.775 (0.623, 0.957) | 0.020 |  | 0.620 (0.432, 0.871) | 0.007 |
|  | Magnesium as categorical variable | 121 / 1072 (11.29%) |  |  | 55 / 1069 (5.15%) |  |  |
|  | Normomagnesemia (0.75-1.02 mmol/L) | 57 / 698 (8.17%) | Ref |  | 21 / 696 (3.02%) | Ref |  |
|  | Hypomagnesemia (< 0.75 mmol/L) | 57 / 329 (17.33%) | 1.563 (1.013, 2.405) | 0.042 | 33 / 328 (10.06%) | 2.673 (1.354, 5.398) | 0.005 |
|  | Hypermagnesemia (> 1.02 mmol/L) | 7 / 45 (15.56%) | 1.482 (0.531, 3.637) | 0.418 | 1 / 45 (2.22%) | 0.548 (0.026, 3.731) | 0.606 |
|  | P for trend |  |  | 0.204 |  |  | 0.005 |
| Sensitivity analysis 3 | Magnesium as continuous (per 1-SD increase) |  | 0.785 (0.638, 0.958) | 0.019 |  | 0.633 (0.456, 0.865) | 0.005 |
|  | Magnesium as categorical variable | 136 / 1110 (12.25%) |  |  | 67 / 1106 (6.06%) |  |  |
|  | [0.75-1.02] mmol/L | 65 / 718 (9.05%) | Ref |  | 27 / 715 (3.78%) | Ref |  |
|  | < 0.65 mmol/L | 29 / 113 (25.66%) | 1.851 (1.052, 3.202) | 0.030 | 21 / 112 (18.75%) | 3.830 (1.730, 8.501) | <0.001 |
|  | [0.65-0.75) mmol/L | 35 / 232 (15.09%) | 1.358 (0.842, 2.160) | 0.202 | 18 / 232 (7.76%) | 1.524 (0.713, 3.186) | 0.267 |
|  | > 1.02 mmol/L | 7 / 47 (14.89%) | 1.275 (0.462, 3.083) | 0.612 | 1 / 47 (2.13%) | 0.440 (0.022, 2.834) | 0.472 |
|  | P for trend |  |  | 0.080 |  |  | 0.001 |
| Sensitivity analysis 4 | Magnesium as continuous (per 1-SD increase) |  | 0.695 (0.470, 0.990) | 0.049 |  | 0.539 (0.254, 1.006) | 0.078 |
|  | Magnesium as categorical variable | 50 / 216 (23.15%) |  |  | 15 / 216 (6.94%) |  |  |
|  | Normomagnesemia (0.75-1.02 mmol/L) | 20 / 122 (16.39%) | Ref |  | 5 / 122 (4.10%) | Ref |  |
|  | Hypomagnesemia (< 0.75 mmol/L) | 27 / 77 (35.06%) | 2.798 (1.422, 5.601) | 0.003 | 9 / 77 (11.69%) | 3.417 (1.067, 12.210) | 0.044 |
|  | Hypermagnesemia (> 1.02 mmol/L) | 3 / 17 (17.65%) | 1.108 (0.235, 3.911) | 0.882 | 1 / 17 (5.88%) | 0.916 (0.045, 6.417) | 0.939 |
|  | P for trend |  |  | 0.022 |  |  | 0.093 |
| Sensitivity analysis 5 | Magnesium as continuous (per 1-SD increase) |  | 0.792 (0.642, 0.971) | 0.026 |  | 0.643 (0.461, 0.881) | 0.007 |
|  | Magnesium as categorical variable | 134 / 1095 (12.24%) |  |  | 65 / 1091 (5.96%) |  |  |
|  | Normomagnesemia (0.75-1.02 mmol/L) | 64 / 708 (9.04%) | Ref |  | 26 / 705 (3.69%) | Ref |  |
|  | Hypomagnesemia (< 0.75 mmol/L) | 63 / 341 (18.48%) | 1.520 (1.004, 2.294) | 0.047 | 38 / 340 (11.18%) | 2.425 (1.285, 4.650) | 0.007 |
|  | Hypermagnesemia (> 1.02 mmol/L) | 7 / 46 (15.22%) | 1.435 (0.509, 3.555) | 0.461 | 1 / 46 (2.17%) | 0.624 (0.030, 4.143) | 0.683 |
|  | P for trend |  |  | 0.190 |  |  | 0.007 |
| Sensitivity analysis 6 | Magnesium as continuous (per 1-SD increase) |  | 0.793 (0.637, 0.981) | 0.035 |  | 0.646 (0.459, 0.894) | 0.010 |
|  | Magnesium as categorical variable | 125 / 1060 (11.79%) |  |  | 58 / 1057 (5.49%) |  |  |
|  | Normomagnesemia (0.75-1.02 mmol/L) | 59 / 689 (8.56%) | Ref |  | 23 / 687 (3.35%) | Ref |  |
|  | Hypomagnesemia (< 0.75 mmol/L) | 59 / 326 (18.10%) | 1.529 (0.995, 2.342) | 0.052 | 34 / 325 (10.46%) | 2.340 (1.230, 4.526) | 0.010 |
|  | Hypermagnesemia (> 1.02 mmol/L) | 7 / 45 (15.56%) | 1.644 (0.577, 4.134) | 0.318 | 1 / 45 (2.22%) | 0.702 (0.034, 4.518) | 0.757 |
|  | P for trend |  |  | 0.264 |  |  | 0.012 |
| Sensitivity analysis 7 | Magnesium as continuous (per 1-SD increase) |  | 0.794 (0.644, 0.975) | 0.029 |  | 0.646 (0.464, 0.884) | 0.008 |
|  | Magnesium as categorical variable | 135 / 1078 (12.52%) |  |  | 67 / 1074 (6.24%) |  |  |
|  | Normomagnesemia (0.75-1.02 mmol/L) | 65 / 696 (9.34%) | Ref |  | 27 / 693 (3.90%) | Ref |  |
|  | Hypomagnesemia (< 0.75 mmol/L) | 63 / 336 (18.75%) | 1.486 (0.983, 2.240) | 0.059 | 39 / 335 (11.64%) | 2.205 (1.184, 4.161) | 0.013 |
|  | Hypermagnesemia (> 1.02 mmol/L) | 7 / 46 (15.22%) | 1.455 (0.519, 3.586) | 0.442 | 1 / 46 (2.17%) | 0.575 (0.028, 3.788) | 0.631 |
|  | P for trend |  |  | 0.215 |  |  | 0.011 |

Sensitivity analysis 1: Patients with fever on admission and leukocytosis within 48 hours were excluded.

Sensitivity analysis 2: Patients with abdominal pain on admission and leukocytosis within 48 hours were excluded.

Sensitivity analysis 3: The magnesium threshold was adjusted and reclassified into four categories.

Sensitivity analysis 4: The analysis was restricted to patients with ascites within 48 hours of admission. Given the relatively small sample size and limited number of outcome events,the multivariable model was adjusted only for the covariates included in Model 2: age, sex, diabetes mellitus, severe liver disease, and the Charlson Comorbidity Index.

Sensitivity analysis 5: Patients who received magnesium supplementation within 48 hours after admission were excluded.

Sensitivity analysis 6: Patients with diarrhea at admission were excluded.

Sensitivity analysis 7: Patients who received SGLT2 inhibitors within 48 hours after admission were excluded.

*Adjusted for age, sex, diabetes mellitus, severe liver disease, Charlson Comorbidity Index, mean arterial pressure, white blood cell count, high-sensitivity C-reactive protein, serum albumin, total bilirubin, serum sodium, international normalized ratio, MELD 3.0 score, loop diuretic use, and potassium-sparing diuretic use.

Abbreviations: HA-SBP, hospital-acquired spontaneous bacterial peritonitis; SGLT2, sodium–glucose cotransporter 2; MELD, Model for End-Stage Liver Disease; SD, standard deviation; OR, odds ratio; CI, confidence interval.

**Figure S1. Percentage of missing data in all variables included in the analysis.** Abbreviations: MELD, Model for End-Stage Liver Disease; HA-SBP, hospital-acquired spontaneous bacterial peritonitis.

**Figure S2. Multicollinearity among covariates was assessed using variance inflation factors.** (A) Hospital-acquired spontaneous bacterial peritonitis. (B) Sepsis. In Model 3, all variables had variance inflation factors less than 5, indicating no significant multicollinearity. Abbreviations: MELD, Model for End-Stage Liver Disease.

**Figure S3. Distribution of the first serum magnesium level within 48 hours after admission.** Serum magnesium levels were normally distributed, with a mean of 0.80 ± 0.13 mmol/L.

**Figure S4. Subgroup analyses of the association between serum magnesium levels and the risk of hospital-acquired spontaneous bacterial peritonitis.** The adjustment variables included age, sex, diabetes mellitus, severe liver disease, Charlson Comorbidity Index, mean arterial pressure, white blood cell count, high-sensitivity C-reactive protein, serum albumin, total bilirubin, serum sodium, international normalized ratio, MELD 3.0 score, loop diuretic use, and potassium-sparing diuretic use. Abbreviations: MELD, Model for End-Stage Liver Disease; OR, odds ratio; CI, confidence interval.

**Figure S5. Predictive performance of serum magnesium for the outcomes.** (A) Hospital-acquired spontaneous bacterial peritonitis. (B) Sepsis.
